# Stem Cells of the Aging Brain

**DOI:** 10.3389/fnagi.2020.00247

**Published:** 2020-08-06

**Authors:** Alexandra M. Nicaise, Cory M. Willis, Stephen J. Crocker, Stefano Pluchino

**Affiliations:** ^1^Department of Clinical Neurosciences and NIHR Biomedical Research Centre, University of Cambridge, Cambridge, United Kingdom; ^2^Department of Neuroscience, University of Connecticut School of Medicine, Farmington, CT, United States

**Keywords:** neural stem cells, aging, senescence, cell metabolism, mitochondria, disease modeling, senotherapeutics

## Abstract

The adult central nervous system (CNS) contains resident stem cells within specific niches that maintain a self-renewal and proliferative capacity to generate new neurons, astrocytes, and oligodendrocytes throughout adulthood. Physiological aging is associated with a progressive loss of function and a decline in the self-renewal and regenerative capacities of CNS stem cells. Also, the biggest risk factor for neurodegenerative diseases is age, and current *in vivo* and *in vitro* models of neurodegenerative diseases rarely consider this. Therefore, combining both aging research and appropriate interrogation of animal disease models towards the understanding of the disease and age-related stem cell failure is imperative to the discovery of new therapies. This review article will highlight the main intrinsic and extrinsic regulators of neural stem cell (NSC) aging and discuss how these factors impact normal homeostatic functions within the adult brain. We will consider established *in vivo* animal and *in vitro* human disease model systems, and then discuss the current and future trajectories of novel senotherapeutics that target aging NSCs to ameliorate brain disease.

## Introduction

Aging is defined as the time-related decline of physiological functions that are necessary for survival eventually leading to the cessation of life (Harman, 2001; López-Otín et al., 2013). The general characteristics of aging, such as cognitive decline, cardiovascular defects, and metabolic changes affect all individuals at varying rates. While some endure aging in a healthy manner, defined as *healthy* aging, others age *unhealthily*, developing age-related disorders such as cardiovascular disease, cancer, Alzheimer’s disease (AD), and diabetes (Chang et al., 2019; Cole et al., 2019). Genetic and environmental factors, such as smoking, exercise, and diet, impart significant alterations to normal physiology that define the rate an organism ages (Harman, 2001). This implies that though two people are the same chronological age their biological brain age may be significantly different (Franke and Gaser, 2019).

Measures have been developed and refined over the preceding decade to assay human brain age *in vivo*, including MRI to both measure gray matter volume and alterations in neural activity. The results from these studies, with due consideration to all caveats and limitations, have demonstrated that a “brain age” can be computed, establishing a novel idea of a “brain age gap.” The “brain age gap” is defined as the gap between a person’s chronological age and their biological brain age based on a pre-determined set of MRI criteria. This computational approach establishes how *healthy* or *unhealthy* an individual’s brain age is and this data can then be extrapolated to then anticipate risk in the development of age-associated brain diseases and can be extended to anticipate the risk of developing age-associated brain diseases (Cole and Franke, 2017). For example, dementia/AD and multiple sclerosis (MS) exhibit the strongest discordance between actual chronological age and predicted age using an unbiased machine MRI learning tool (Kaufmann et al., 2019). Typically, the effects of advancing chronological age, including cognitive decline, become significantly apparent between the ages of 60–75 years as evidenced by the significant loss of total tissue volume, which coincides with the peak onset of age-associated neurodegenerative diseases (Scahill et al., 2003; Chad et al., 2018; Franceschi et al., 2018). These diseases can also occur earlier in life in subjects with higher genetic and environmental risk factors, which suggests an accelerated aging phenotype may play a role. Similar deficits in cognitive function correlated with changes in brain volume are observed in aged humans and rodents (20–24 months old), making them a valuable model (Hamezah et al., 2017). Furthermore, most of these morphological changes are correlated with increased synaptic dysfunction, which leads to the gain-of-function of behavioral deficits observed in aging, such as cognitive decline, learning, memory, and sensory deficits (Petralia et al., 2014). Both human and rodent work has suggested a correlation between a decrease in the number of synaptic connections and an increase age-related cognitive decline (Dickson et al., 1995; Peters et al., 2008). The predominant hallmark used to compute biological age in humans is brain volume, which is associated with a decrease in synaptic connections, suggesting a lack of renewal, replacement, and regeneration on a cellular level.

Neural stem cells (NSCs) persist throughout mammalian life, residing within the subgranular zone (SGZ) of the hippocampus and the subventricular zone (SVZ) of the lateral ventricles, where they maintain the capacity for self-renewal and maturation into new neurons and glia. NSCs are mitotic cells characterized by symmetric divisions (self-renewal) during early development. They gradually shift to asymmetrical division to generate differentiated cells and maintain a multipotent reservoir (Gage, 2000; Alvarez-Buylla and Lim, 2004; Zhao et al., 2008; Gage and Temple, 2013; Obernier and Alvarez-Buylla, 2019). Although the proliferation and differentiation of NSCs are predominantly restricted to the embryonic period in rodents, the capacity to produce new neurons has been shown to persist into adulthood, however, whether this is an evolutionarily conserved process in humans remains controversial (Sorrells et al., 2018; Moreno-Jiménez et al., 2019). Evidence for neurogenesis in healthy adult humans is provided by the presence of NSCs and immature neurons both expressing cell division markers (Moreno-Jiménez et al., 2019; Tobin et al., 2019). Also, neurogenesis has been reported using radioactive carbon-based cell dating and BrdU incorporation studies (Eriksson et al., 1998; Spalding et al., 2013). These dynamics in humans have been observed to change with disease, such as AD, where immature neurons are found greatly reduced, and MS, where NSCs are found increased in the SVZ (Nait-Oumesmar et al., 2007; Moreno-Jiménez et al., 2019).

In adult mice, NSCs residing within the specialized niches are known to play important roles in maintaining cognitive functions, such as learning and memory formation, and contributing to repair and regeneration of injured tissue, which includes their neurogenic capability (Imayoshi et al., 2008; Lugert et al., 2010). These new neurons contribute to learned behavior such as odor reward association and discrimination (Grelat et al., 2018; Li et al., 2018). Advancing age-related biological changes are associated with a significant decline in neurogenesis, concomitant with dynamic alterations to the niche microenvironment that disrupt normal homeostatic functions (Kuhn et al., 1996).

**Figure 1:**
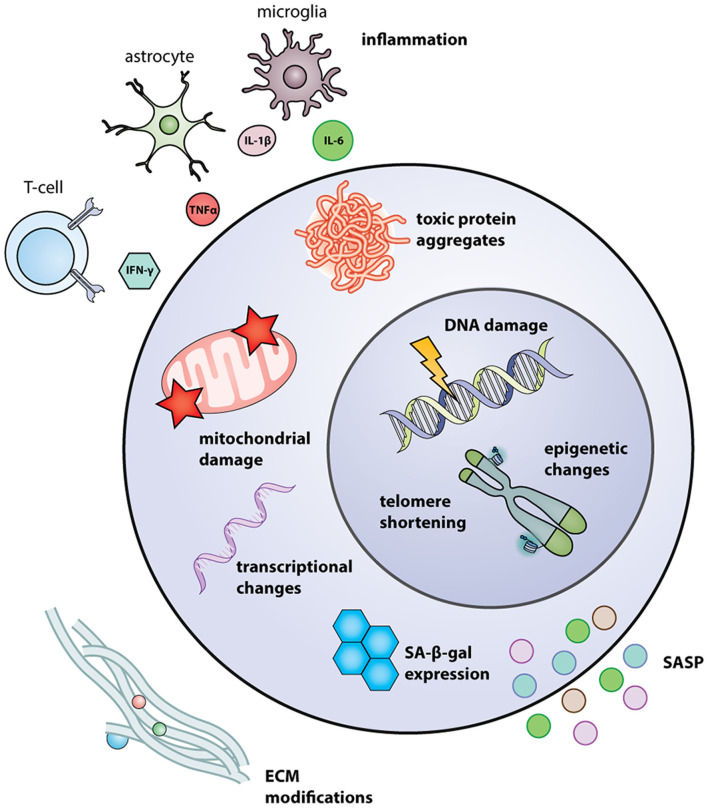
Intrinsic and extrinsic regulators of NSC aging. Intrinsic regulators include transcriptional changes, such as upregulation of p16^Ink4a^ and p53; mitochondrial damage, resulting from mtDNA mutations and excessive ROS; toxic protein aggregates, due to a dysregulation in proteostasis; SA-β-gal expression, induced by senescence; telomere shortening and DNA damage caused by replication-associated stress and dysfunctional repair amongst other factors; and changes in epigenetic patterns. Extrinsic regulators include SASP, secreted by nearby senescent cells, and secreted by senescent NSCs themselves; modifications in ECM, such as increases in HA composition; and inflammatory molecules secreted by activated astrocytes, microglia, and T-cells. Abbreviations: ECM, extracellular matrix; HA, hyaluronic acid; mtDNA, mitochondrial DNA; NSC, neural stem cells; ROS, reactive oxygen species; SASP, senescence-associated secretory phenotype.

Several tissues and cell-based biological biomarkers have been identified that reflect age-related processes, such as senescent cell burden, genomic instability, on-going chronic inflammation, and telomere attrition (Figure 1; López-Otín et al., 2013). The accumulated burden of aging defects contributes to overall brain degeneration, drastically impacting the regenerative potential of the NSC niches. Also, ongoing disease burden has been shown to accelerate biological brain aging in AD/dementia, PD, amyotrophic lateral sclerosis (ALS), MS, and Huntington’s disease (HD), all neurodegenerative diseases with demonstrated dysfunction in NSC compartments (Horgusluoglu et al., 2017; Hou et al., 2019).

In this review article, we will highlight how intrinsic, as well as extrinsic regulators of age and disease, impart drastic changes to the endogenous NSC niche, how this is modeled *in vitro* and *in vivo*, and new therapies on the horizon targeted at aging NSCs to ameliorate brain disease.

## Brain Stem Cell Niches in Aging and Disease

Mammalian NSCs within niches adopt various states including quiescence, activation, and differentiation, however, the proportions of cells in those states change dramatically with increasing biological age (Obernier and Alvarez-Buylla, 2019). A large body of evidence has revealed that aging negatively impacts these niches, ultimately impacting regenerative functions. One major component being cellular senescence, which is a process in which cells experience a decrease in their proliferative capacity and undergo profound alterations which inherently changes their normal functions, impacting surrounding cells, and eventually leading to age-associated organ dysfunction (Baker and Petersen, 2018). Senescent cells accumulate with age and impact regeneration and homeostatic functions within stem cell niches, and experimental evidence in mice has linked the elimination of these cells to an increase in lifespan and a delay in age-associated disorders (Baker et al., 2011; Yousefzadeh et al., 2020). They disturb the microenvironment by secreting a wide-range of factors, including pro-inflammatory proteins, chemokines, and interleukins (ILs) which have a profound effect on other cells around them, termed the senescence-associated secretory phenotype (SASP; Coppé et al., 2010; Angelova and Brown, 2019; Cohen and Torres, 2019). Senescent cells have been identified in the aging brain of both humans and rodents, particularly in the context of neurodegenerative disease (Baker and Petersen, 2018). Senescence is initiated by a myriad of aging-related factors, both intrinsic and extrinsic, which will be reviewed in this section.

### DNA Damage and Repair

Several studies have demonstrated that chronological aging results in the increased accumulation of DNA damage in tissues and cells (Kenyon, 2010; Sperka et al., 2012). For example, replication-associated DNA damage leads to mutations and increases genomic instability with advancing age (Adams et al., 2015). In line with this observation, NSCs isolated from the SVZ of 20-month old-aged mice displayed significant genetic aberrations compared to NSCs isolated from 2-month old mice, which is attributed to the increased accumulation of mutations over replicative cycles (Bailey et al., 2004). NSCs avoid replication stress by entering an inactive stage called quiescence, highlighted by a low basal metabolic rate and limited self-renewal. Following disease or tissue damage, reactive oxygen species (ROS) production stimulates adult NSCs to re-enter the cell cycle to replenish damaged and dying cells. Coincidentally, this results in replication-induced stress and an increased frequency of DNA mutations (Wang Y. Z. et al., 2011). This highlights the importance of maintaining a proper balance between quiescence and activation within NSC niches to minimize replicative stress and maximize DNA integrity during chronological and biological aging.

**Figure 2:**
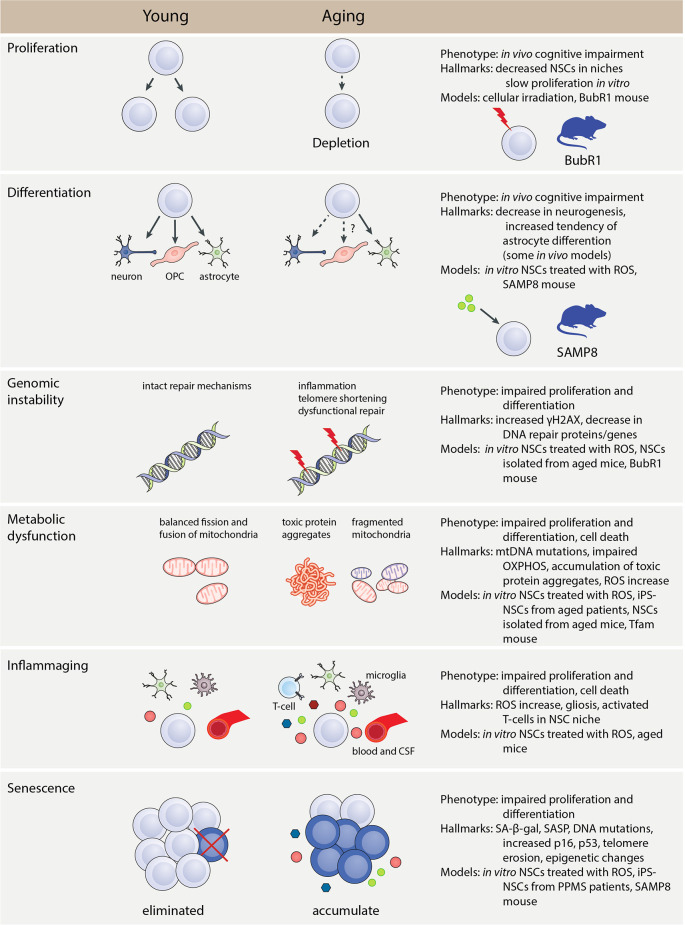
Hallmarks and phenotype of NSC aging. The main phenotypes, hallmarks, and models that can be used to investigate aspects of NSC aging. Genomic instability, metabolic dysfunction, inflammaging, and senescence are all aspects that affect the normal proliferation and differentiation function of NSCs. These causes of aging have specific hallmarks that can be identified using *in vivo* and *in vitro* methods. Abbreviations: CSF, cerebrospinal fluid; iPSC, induced pluripotent stem cell; mtDNA, mitochondrial DNA; NSC, neural stem cell; OPC, oligodendrocyte progenitor cell; OXPHOS, oxidative phosphorylation; PPMS, primary progressive multiple sclerosis; ROS, reactive oxygen species; SA-β-gal, senescence-associated β-galactosidase; SASP, senescence-associated secretory phenotype.

Genomic instability with advancing age is thought to be the result of increased accumulation of DNA damage in tandem with a diminished DNA damage response. It has been reported that NSCs fail to proliferate and undergo appropriate differentiation in the absence of functional DNA repair mechanisms (Figure 2; Marin Navarro et al., 2020). Under normal non-aged conditions cells still face genotoxic insults from endogenous by-products such as ROS, inflammatory mediators, and lipid peroxidation while maintaining DNA damage response and repair processes (Mani et al., 2020). This includes the five major repair pathways, base excision repair (BER), nucleotide excision repair (NER), mismatch repair (MMR), homologous recombination (HR), and non-homologous end joining (NHEJ), which all involve a complex interplay of genes and their ensuing proteins. These pathways attempt to repair the lesioned area, however, breakdowns in signaling activate apoptotic mechanisms to inhibit the propagation of the lesioned DNA (Petr et al., 2020). The efficiency in these processes declines with age for reasons that remain unclear leading to a build-up of DNA damage. Evidence points to transcriptional repression of DNA repair genes in aging cells as well as age-related decreases in repair enzymes and activities accounting for the increased DNA damage with age (Szczesny et al., 2003; Imam et al., 2006; Collin et al., 2018). Additionally, DNA damage response elements such as NER, BER, MMR, and NHEJ are important in the maintenance of the NSC niche with defective elements associated with age-related neurodegenerative diseases such as ALS, AD, and PD (Liu and Martin, 2006; Sepe et al., 2016;Shanbhag et al., 2019).

Retrotransposable elements (RTEs) are DNA elements that have been copied and pasted from one location to another and can also give rise to genomic instability through mutations and double-strand breaks which in turn impacts gene regulation (Terry and Devine, 2019). Further, increased activity of RTEs has been observed in the aged human brain and results in genome instability in NSCs (Bollati et al., 2009; Maxwell et al., 2011). One example being long interspersed nuclear element-1 (LINE-1), which is normally repressed by *Sox2*, a gene required for the maintenance of NSCs with diminished expression in the hippocampal SGZ with advancing age (Muotri et al., 2005; Carrasco-Garcia et al., 2019). Decreased expression of *Sox2* within the hippocampal SGZ in aged mice is inversely correlated with the increased expression of the cyclin-dependent kinase inhibitor p16^Ink4a^, an established biomarker of senescent cells (Carrasco-Garcia et al., 2019). Specifically, LINE-1 becomes transcriptionally derepressed in p16^Ink4a^ expressing cells, which triggers a type-I interferon (IFN-1) response and initiates the secretion of pro-inflammatory cytokines, proteases, and immune modulators inhibiting NSC proliferation and leading to age-associated chronic inflammation (Coppé et al., 2010; Kulkarni et al., 2017; De Cecco et al., 2019).

Genomic instability can also result from telomere shortening, one of the major mechanisms proposed to induce organismal aging (López-Otín et al., 2013). Telomeres are specialized structures that cap the ends of chromosomes protecting from gene erosion and fusion with neighboring chromosomes (Sahin and Depinho, 2010). In humans, the average telomere length declines in advancing age due to truncation occurring following a lifetime of cell division, with the endogenous enzyme telomerase maintaining telomere length to protect against genomic instability and intrinsic DNA damage (Arai et al., 2015). Importantly, adult NSCs within the rodent SVZ are known to have high telomerase activity to meet the proliferative and self-renewal needs of the cell (Caporaso et al., 2003), however telomerase in isolation cannot maintain telomere length within NSC niches. In fact, the deleterious effects of shortened telomeres in NSCs are mediated, in part, by p53, a tumor suppressor protein and potential biomarker of aging and senescence with increased expression in aged tissues (Rodier et al., 2007). Mutant mice overexpressing p53 have a reduction in NSC proliferation, highlighting a role for p53 in the aging NSC niche (Medrano et al., 2009). When considered together, this work underscores the importance of genomic stability in the maintenance and proper functioning of adult NSCs (Figure 2).

### Intrinsic Regulators

The frequency of intrinsic regulators of DNA damage increases with advancing age, negatively influencing the expression of regulatory genes important for homeostasis (Figure 2; Espada and Ermolaeva, 2016). For example, members of the forkhead box transcription factor family O (FoxO) are indispensable in maintaining NSC proliferation and self-renewal throughout adulthood by regulating a program of genes that controls quiescence, differentiation, and oxygen metabolism (Paik et al., 2009; Renault et al., 2009). Constitutive knock-out of *Foxo3* in mice reduces the total NSC pool and leads to decreased self-renewal and impaired ability to generate different neural lineages (Renault et al., 2009). Supporting this notion, diminished regulatory gene expression either indirectly or directly underlies NSC dysfunction in advancing age. A follow-up study identified an alternative function of *Foxo3* in directly regulating autophagy genes in NSCs with its loss leading to the accumulation of protein aggregates and resultant homeostatic dysfunction (Audesse et al., 2019). This is of particular importance as protein aggregation is a key histological hallmark in many age-related neurodegenerative diseases, such as the accumulation of amyloid-β (Aβ) and tau tangles in AD and alpha-synuclein deposits in PD. *Foxo3* remains an attractive target for further functional studies and therapeutic targeting as genome-wide association studies have identified a single-nucleotide polymorphism (SNP) within the *FOXO3* gene that is associated with extreme longevity in humans (Willcox et al., 2008; Flachsbart et al., 2009).

Intrinsic DNA damage also occurs in mitochondrial (mt)DNA. A higher incidence of mtDNA mutations is known to be associated with physiological brain aging and in age-related neurodegenerative diseases such as AD and PD (Zapico and Ubelaker, 2013; Cha et al., 2015). Somatic mtDNA mutations accumulate in all tissues with age in a stochastic manner (Chinnery et al., 2002; Bratic and Larsson, 2013). It is still unclear why somatic mutations in mtDNA increase with age, but most evidence points to increased pro-inflammatory insults (Larsson, 2010). An alternative hypothesis implicates replication errors that occur during embryogenesis which undergo expansion in adult life and accumulate (Larsson, 2010). Therefore, further studies investigating the cause of mtDNA mutations in aging are warranted.

Once an arbitrary threshold is surpassed, where the levels of accumulated mtDNA mutations surpass normal/healthy mtDNA, energy metabolism is impaired (Figure 2; Wallace, 2010). This is significant in tissues with high energy demands such as the NSC niches of the developing and injured adult brain (Cavallucci et al., 2016). mtDNA integrity is maintained *via* repair machinery such as 8-oxoguanine DNA glycosylase (OGG1), a DNA repair protein that restores damaged mtDNA. This repair machinery is important in maintaining NSC multipotency as evidenced in NSCs from *Ogg1* knockout-mice which spontaneously accumulate mtDNA damage, shifting their differentiation pattern towards an astrocytic lineage vs. neuronal (Wang W. et al., 2011). Further, two separate reports have identified a decrease in human *OGG1* activity in the AD brain (Mao et al., 2007; Jacob et al., 2013). mtDNA is also susceptible to damage due to a lack of histone protection and proximity to ROS generated by mitochondria (Alexeyev et al., 2013). An overabundance of ROS in NSC niches impairs adult neurogenesis (Rola et al., 2007). The accumulation of mtDNA mutations over time results in respiration deficiency and the further, excessive production of ROS (Murphy, 2009). Mitochondrial ROS act as regulators to facilitate gene expression changes that impact NSC fate decisions (Maryanovich and Gross, 2013). Ablating the mitochondrial transcription factor A (*Tfam*) in mice produces histological and pathological hallmarks of aging in the hippocampus of mice, including impaired proliferation and neurogenesis in NSCs (Beckervordersandforth et al., 2017a). Interestingly, reductions in the TFAM gene and protein expression are observed in advancing age and age-associated neurodegenerative diseases such as PD, AD, and HD (Kang et al., 2018). Overall, these studies point to the natural accumulation of mtDNA mutations in normal aging, which may be accelerated in neurodegenerative disorders, leading to widespread NSC dysfunction.

### Epigenetics

Epigenetic modifications naturally occur and accumulate with age resulting in long-lasting functional changes to cells and tissues (Figure 1). These modifications are attributed to cell-intrinsic and extrinsic factors inherited through subsequent cell divisions (Feil and Fraga, 2012; Podobinska et al., 2017). An example of this is histone acetylation and deacetylation, which is regulated by histone acetyltransferase (HAT) and deacetylase (HDAC) enzymes, respectively. Acetylation of histones relaxes the chromatin structure increasing accessibility to the gene for transcription. In aged mice, an imbalance in the acetylation and deacetylation of genes in the hippocampus is correlated with memory impairment (Peleg et al., 2010). Epigenetic modifications also occur through DNA methylation, a crucial process regulating and controlling gene expression by various DNA methyltransferases (DNMTs). Changes in the DNA methylation status are observed in the brain of human post-mortem subjects with advanced age (Numata et al., 2012). Interestingly, the biological aging of tissue correlates with patterns in DNA methylation termed the “epigenetic clock” (Horvath et al., 2012). Tet methylcytosine dioxygenase 2 (TET2), a regulator of DNA methylation, is reduced in human aging and somatic mutations are associated with an elevated risk of developing age-related disorders such as stroke (Burgess, 2015). Similarly, Tet2 expression is reduced in NSCs from aged rodents with complete ablation inhibiting normal neurogenesis (Gontier et al., 2018). Many different epigenetic factors are known to alter adult NSC renewal and differentiation capacity, such as mixed-lineage leukemia 1 (Mll1), a chromatin remodeling factor identified in mice, yet their role in age and age-associated diseases remain to be elucidated (Lim et al., 2009).

Nucleoporins (Nups) are a family of proteins that form channels within the nuclear envelope, thus regulating the flow of macromolecules, and have emerged as epigenetic regulators of NSC differentiation (Raices and D’Angelo, 2017). Differentiation of NSCs is accompanied by changes in nuclear reorganization which result in gene expression changes mediated by Nup expression (Capelson et al., 2010). Furthermore, Nups physically interact with the genome to control transcriptional programs. In NSCs, NUP153 is essential for maintaining stemness *via* its complex with the transcription factor *Sox2*. In turn, the silencing of *Nup53* results in neuronal differentiation, associated with a loss of *Sox2* expression (Toda et al., 2017). NUP153 has also been implicated in age-related diseases, such as AD (Sakuma and D’Angelo, 2017; Leone et al., 2019). In NSCs isolated from the 3xTg-AD mouse model, *Nup153* expression was significantly reduced, inhibiting proliferation, migration, and neuronal differentiation. Instead, *Nup153* overexpression in AD NSCs rescued defective proliferation and migration and enhanced neuronal differentiation. Thus, *Nup153* acts as an epigenetic hub for a variety of co-regulators (Leone et al., 2019). Different outcomes in NSC differentiation associated with *Nup153* expression may also be context dependent and in turn change the epigenetic behavior of *Nup153*. A further understanding in how Nups alter NSC behavior in aging and disease is warranted.

### Cell Metabolism

The metabolism of stem cells plays a vital role in maintaining cellular homeostasis through the regulation of various processes, such as the generation of energy and proteins along with the breakdown and elimination of waste. Changes in the balance of these functions alter NSC behavior, especially the propensity to proliferate and differentiate (Figure 2). Many of these basic homeostatic functions, such as mitochondrial maintenance, proteostasis, and nutrient sensing, are altered with age and considered hallmarks of aging (López-Otín et al., 2013). Rapamycin, a drug that targets the mammalian target of rapamycin (mTOR) pathway, which is critical to cellular metabolism, has been identified to prolong rodent lifespan, further elucidating the importance of cellular metabolism in aging (Harrison et al., 2009). Probing the mechanisms by which aging alters the variety of metabolic aspects is important in the discovery of new avenues for therapeutic targeting.

Considerable activity into understanding the biological processes that modify NSC metabolism in advancing age have demonstrated the importance of proteostasis, or protein homeostasis, in maintaining cellular health. During advancing age, breakpoints in proteostasis result in the accumulation of misfolded proteins and associated cellular damage and dysfunction (Figure 1; Koga et al., 2011). Several neurodegenerative diseases are identified by the accumulation of misfolded and damaged proteins, such as Aβ in AD, alpha-synuclein deposits in PD, and Lewy bodies in dementia, which are known to negatively affect NSC niches (Basaiawmoit and Rattan, 2010). NSCs from aged mice have been shown to accumulate protein aggregates in defective lysosomes, limiting normal digestion and removal in quiescent NSCs of aged rodents. Lysosomal dysfunction, and in turn accumulation of insoluble protein aggregates, prevented quiescent NSCs from aged animals to return to an activated, proliferative state. Enhancing lysosomal function through transcription factor EB (TFEB) activity, a master regulator of lysosomes restores NSC activation ability (Leeman et al., 2018). A functional outcome of damaged protein accumulation in NSCs with advancing age is a weakening of the diffusion barrier which maintains the distribution of cellular components during replication or differentiation. This prevents asymmetric segregation of damaged proteins and further propagates the accumulation of misfolded proteins in daughter cells (Moore et al., 2015). Also, accumulation of protein fragments in neurodegenerative diseases such as AD, PD, and HD alters normal NSC function and can promote a senescent phenotype (Conforti et al., 2013; He et al., 2013; Zasso et al., 2018).

Changes in NSC metabolism help balance self-renewal and differentiation, which includes mechanisms involved in energy generation (Rafalski and Brunet, 2011; Ochocki and Simon, 2013). A major emphasis has been placed on contextualizing mitochondrial dysfunction and NSC aging and whether it supports the *mitochondrial theory of aging*, which proposes that chronic mitochondrial dysfunction underlies the great majority of age-related deficits (López-Otín et al., 2013). This theory is not without merit, as the capacity of antioxidants to scavenge ROS in the brain is impaired with age, allowing the extra- and intracellular accumulation of ROS and subsequent oxidative stress and damage (Siqueira et al., 2005). Quiescent NSCs are intrinsically glycolytic unless activated for proliferation wherein a metabolic switch to mitochondrial respiration *via* oxidative phosphorylation (OXPHOS) occurs to meet the new energy demands of the cell (Coller, 2019). However, aging impairs mitochondrial respiration through a variety of factors that damages the machinery necessary for controlled activation of NSC proliferation and differentiation, directly impacting adult neurogenesis (Figure 2; Llorens-Bobadilla et al., 2015; Stoll et al., 2015). For example, conditional deletion of the energy-producing mitochondrial enzyme complex alpha-ketoglutarate-dehydrogenase reduces the proliferative potential of NSCs residing in the SGZ of the hippocampus (Calingasan et al., 2008). Alterations in other mitochondrial properties, such as fusion and fission, impair NSC proliferation in the SGZ of the adult rodent hippocampus leading to deficits in spatial learning (Khacho et al., 2016). Additionally, stem cells upon division apportion aged mitochondria, leaving a daughter cell with an accumulation of aged, fragmented mitochondria causing a deficit in stemness properties (Katajisto et al., 2015). Therefore, normal mitochondrial function is critical in maintaining healthy NSC niches, allowing NSCs to react appropriately to the changing energy needs of the brain.

Nutrient sensing by NSCs within niches is inherently linked to the regulation of their activation state. Recently, mechanisms through which calorie restriction promotes longevity in NSCs have been uncovered (Van Cauwenberghe et al., 2016). Calorie restriction prevents age-related declines in neurogenesis, which likely occurs *via* a decrease in pro-inflammatory signaling in the niche (Apple et al., 2019). The effects of calorie restriction are partly mediated through the insulin/insulin-like growth factor (IGF)-1 signaling (IIS) pathway which responds to changes in glucose and is found to regulate NSC function (Rafalski and Brunet, 2011; Vitale et al., 2019). Inhibition of IGF-1 signaling in rodent NSCs inhibits age-related declines and promotes self-renewal of the NSC niche (Chaker et al., 2015). Intriguingly, signaling through the IIS pathway results in the downstream activation of mTOR, a pathway with major implications in aging (Verburgh, 2015). The mTOR pathway acts as a nexus in amino acid concentration signaling and is a critical regulator of cellular metabolism and growth. mTOR signaling is important in regulating the quiescent vs. an active proliferative state of adult NSCs (Nieto-González et al., 2019). For example, inhibition of mTOR complex 1 (mTORC1) induces a reversible quiescent phenotype, which is corroborated by *in vivo* findings that diminished mTOR activity in the aged NSC niche can be reversed by pharmacological intervention leading to increased proliferation and neurogenesis (Paliouras et al., 2012; Romine et al., 2015). However, future studies must proceed with caution when artificially increasing and decreasing mTOR activity. The mTOR pathway is hyperactivated in both pre-clinical rodent models of AD and in AD patients with associated NSC dysfunction, suggesting that finely-tuned mTOR activity is needed to maintain niche homeostasis (Perluigi et al., 2015). In this regard, recent evidence has identified reductions in mTOR activity *via* rapamycin supplementation concomitant with the amelioration of age-dependent spatial memory deficits and increased lifespan in mice (Majumder et al., 2012). Whether rapamycin treatment has an indirect or direct role in modulating the aged NSC niche has yet to be tested.

Physical exercise has a positive effect on cognitive functions in older adults, also reflected in rodent research (Colcombe et al., 2006). It has been suggested that aerobic exercise boots aspects of brain metabolism, including upregulation in the hippocampus of enzymes involved in glutamate turnover and glucose catabolism (Ding et al., 2006; Matura et al., 2017). This results in enhanced proliferation of NSCs and increases in the number of immature neurons in middle-aged mice (Wu et al., 2008). Voluntary running in aged mice increases secretion of growth hormone stimulating proliferation and neurogenesis in the hippocampal niche of aged mice (Yang et al., 2015). Moreover, increases in BDNF and IGF-1 significantly increase after voluntary running, improving neurogenic potential (Adlard et al., 2005). This data suggests that exercise aids in the proliferation of NSC niches, and the eventual production of immature neurons through overall changes in metabolic components.

### Oxygen Sensing

Oxygen sensing is a potent regulator of the aged NSC niche. Hypoxia-inducible factors (HIFs) are transcription factors that regulate the cellular response to changes in oxygen levels, of which HIF1α plays a prominent role in NSCs. Conditional deletion of HIF1α in NSCs of adult mice resulted in a loss of NSC proliferation within the SVZ, highlighting its importance in maintaining the adult NSC niche (Li et al., 2014). Further, chronic mild hypoxia results in dual yet opposing NSC functions. Chronic mild hypoxia increases NSC proliferation in the presence of mitogens such as EGF and bFGF, whereas chronic mild hypoxia promotes differentiation into neurons in the absence of these mitogens (Xie and Lowry, 2018). Hypoxia is known to play a role in multiple neurodegenerative diseases, including AD, PD, ALS, and HD, where it promotes inflammation and neuronal damage. However, the extent of the alterations to the NSC niche is unknown (Jha et al., 2018). Impaired oxygen sensing is reported in aged rodent brains suggesting a potential mechanism leading to neurogenic decline with age (Yeo, 2019).

### Extracellular Matrix

It is well-established that the ECM is an important component of the NSC niche serving as an inert scaffold to anchor cells and dynamically regulate cell fate (Figure 1; Gattazzo et al., 2014). NSC adherence to ECM molecules such as laminin (Ln), fibronectin (Fn), and hyaluronan (HA) is facilitated *via* integrin receptors, which initiate cellular responses stimulating proliferation or differentiation. Concerning advancing age, Su et al. (2017) demonstrated that increased HA deposition within the dentate gyrus (DG) directly contributes to age-related reductions in NSC proliferation and differentiation by overactivation of the CD44 receptor. Further, increased HA deposition is observed in aged human brain tissue and in demyelinated MS plaques, the latter impeding remyelination (Back et al., 2005; van Horssen et al., 2007; Lepelletier et al., 2017). Stem cell behavior is also dependent on tissue stiffness, which is determined by ECM composition and organization. ECM-derived mechanical signals act through Rho GTPases to activate cellular pathways to alter NSC lineage commitment. When NSCs are cultured on stiff matrices neurogenesis is suppressed whereas blocking Rho GTPase in NSCs rescues differentiation on stiff matrices (Keung et al., 2011). Follow-up studies identified that soft hydrogels direct cultured adult NSCs to differentiate into neurons, whereas harder gels were biased towards glial cell differentiation (Saha et al., 2008). Counterintuitively, despite overall brain stiffness increasing with age in both rodents and humans (Elkin et al., 2010; Takamura et al., 2020), decreased stiffness in neurodegenerative diseases is linked to a loss of adult neurogenesis (Klein et al., 2014). These differences could be due to regional heterogeneity in brain stiffness, which is observed in post-mortem tissue of AD and PD patients, highlighting the complexity of ECM regulation and deposition in the aging and diseased brain (Berg, 2011; Murphy et al., 2016). Moreover, the presence of glial scar tissue and brain tumors can drastically change the mechanical properties of the affected brain region in near an NSC niche and, in turn, the behavior. Thus, the ECM plays a major role in modulating the activity of the NSC niche in both aging and disease, although the extent to which it drives total brain aging, and perhaps neurodegeneration, remain outstanding lines of research.

### Extrinsic Regulators, Immunity, and Inflammation

Key drivers in the development of an aged phenotype in adult NSCs involve extrinsic large-scale systemic changes reflected in circulating fluids, including blood, cerebrospinal fluid (CSF), and lymph which can affect the NSC niche (Figure 1). The blood, CSF, and lymph display increases in pro-inflammatory mediators with both age and neurodegenerative disease exacerbating ongoing brain-wide inflammation and NSC niche dysfunction (Pluvinage and Wyss-Coray, 2020). Recent work has uncovered alterations in the human plasma proteome with age, which become exacerbated with AD (Lehallier et al., 2019). Further study in what these proteomic changes biologically confer is warranted.

Due to the SVZ being uniquely situated above the lateral ventricles, exposure to blood and CSF factors routinely occurs. Systemic factors have been identified in aged mice that promote age-induced dysfunction, such as C-C motif chemokine 11 (CCL11), a chemokine increased in the blood of aged mice which directly correlates with a decrease in neurogenesis. Systemic exposure of young mice to recombinant CCL11 impaired hippocampal neurogenesis, which is rescued with a CCL11 blocking antibody. Interestingly, CCL11 is increased in aged human plasma and CSF (Villeda et al., 2011). β2-microtubulin (B2M) is another circulating pro-aging factor identified in old mice as well as humans. It negatively regulates regenerative functions in the hippocampus, including NSC proliferation and neurogenesis (Smith et al., 2015).

On the other hand, aged mice with cognitive deficits show improvement after parabiosis with young mice, highlighting how circulating systemic factors can directly influence brain homeostasis (Villeda et al., 2011, 2014). For example, several circulating factors have been identified in young mice which provide rejuvenation to aged NSC niches, one being growth differentiation factor 11 (GDF11), a bone morphogenetic protein. This protein was found to be present in higher concentrations in young mouse serum compared to aged mouse serum. Also, after heterochronic parabiosis of aged mice to young mice, GDF11 was found increased in the aged mice. Administration of a recombinant version of GDF11 to aged mice reproduces similar beneficial effects, such as increased NSC proliferation and differentiation, as observed in the parabiosis studies (Katsimpardi et al., 2014). Further work delving into an earlier developmental stage found that umbilical cord plasma from humans contains proteins that improve cognitive function in aged mice, namely tissue inhibitor of metalloproteinases 2 (TIMP2). TIMP2 is enriched in human cord plasma, young mouse plasma, and in young mouse hippocampi, and declines with age. Immunodepletion of TIMP2 from cord plasma before intravenous administration into aged mice blocked the beneficial cognitive effects emphasizing the importance of systemic factors in brain aging (Castellano et al., 2017).

The blood-brain barrier (BBB) is comprised of a microvasculature unit of brain endothelial cells (BECs), which are exposed to circulating factors on their luminal side and brain cells on their abluminal side. BECs in aged mice express an inflammatory transcriptional profile, including upregulation of vascular cell adhesion molecule 1 (VCAM1). Soluble VCAM1 is increased in the plasma of both aged humans and mice. Plasma collected from aged mice and infused into young mice induced a significant increase in VCAM1 expression in the BEC population, which caused microglial activation and decreased the amount of proliferating NSCs. Genetic deletion of *Vcam1* in BECs during adulthood reduced microglial activation and increased NSC proliferation (Yousef et al., 2019). Given that aged BECs display a pro-inflammatory profile, exposure to circulating pro-inflammatory factors may further amplify this phenotype through induction of VCAM1 expression as well as induce intrinsic cellular changes associated with aging.

Aging and neurodegenerative diseases are associated with impaired integrity of the BBB, which creates a more permissive environment for the entry of peripheral immune cells into the brain parenchyma (Erdö et al., 2017). Infiltrating T cells secreting high levels of IFN-γ have been identified in aged NSC niches. Here, increased IFN-γ led to impaired NSC proliferation and maintenance of a quiescent phenotype, impeding differentiation. Markedly, histological analysis of post-mortem human brain tissue from elderly individuals identified a similar pro-inflammatory T cell infiltrate in the SVZ (Dulken et al., 2019). The aberrant infiltration of T cells into the SVZ in aged mice is supported by previous work demonstrating that under chronic inflammatory conditions, such as in the rodent model of MS, experimental autoimmune encephalomyelitis (EAE), an autoreactive T-cell-mediated disease model, T cell activation is associated with a dysfunction in the proliferation and migration of NSCs due to persistent inflammation (Pluchino et al., 2008; Rasmussen et al., 2011).

The term *inflammaging* is used to describe major changes in aging associated with the accumulation of soluble inflammatory factors, tissue damage burden, and activation of immune cells (López-Otín et al., 2013). Overall, the above work supports the concept that pro-inflammatory molecules from circulating fluids as well as infiltrating cells affect the NSC niche (Figure 1). Progenitor cells in the brain also have the capability of secreting pro-inflammatory factors. One example being senescent stem cells in progressive MS brains secreting high mobility group box 1 (HMGB1), a known SASP factor and inflammatory molecule, which inhibits the differentiation of oligodendrocyte progenitor cells (OPCs) *in vitro* (Davalos et al., 2013; Nicaise et al., 2019). HMGB1 has previously been found to be elevated in the hippocampus and CSF of aged rats where it promotes microglial inflammation by stimulating toll-like receptors (TLR)-2 and -4 (Fonken et al., 2016). Inflammatory factors can also be secreted by brain cells within the niche microenvironment. A recent article highlighted the impact of age-associated changes within the niche, including the activation of microglia, the resident brain macrophage population (Solano Fonseca et al., 2016). Increased clusters of activated microglia and peripheral infiltrating monocytes expressing increased levels of tumor necrosis factor-α (TNF-α), interleukin-1 β (IL-1β), and IL-6 were identified in the aged SVZ while increased levels of TGF-β1 were found to be released from SVZ endothelial cells (Pineda et al., 2013). *In vitro* modeling demonstrated that resting, non-activated microglia promote NSC proliferation and differentiation into neuronal lineages, whereas activated pro-inflammatory microglia suppressed differentiation (Solano Fonseca et al., 2016). The pro-inflammatory cytokine IL-1β, which is released by activated microglia and astrocytes, increases VCAM1 expression in NSCs repressing them in a quiescent state, inhibiting neurogenesis, and diminishing expansion of the NSC pool through self-renewal (Kokovay et al., 2012). Microglia are not the only contributors to *inflammaging*. Astrocytes become reactive with advancing age wherein they secrete pro-inflammatory molecules (e.g., IFNs) directly impacting local cell networks (Clarke et al., 2018). Corroborating data from animals lacking global expression of IFN receptors demonstrate an absence of age-related declines in NSC activation; rather, NSCs readily activate from quiescence as observed in young animals (Kalamakis et al., 2019). Unsurprisingly, an increased response to IFN is detected in the aged human brain which is thought to negatively affect brain functions (Baruch et al., 2014). Overall, these data suggest that chronic inflammation in the brain, whether a result of age, on-going neurodegenerative disease activity, or a combination of both, maintains NSCs in a quiescent state, repressing their capacity to proliferate and differentiate (Figure 2).

These findings demonstrate that both systemic and local factors are altered with age and disease, as well as widespread accumulation of senescent cells, leading to profound impacts on NSCs and niche microenvironment. However, it remains to be understood how alterations in the absolute amounts of these factors in advancing age and age-related neurodegenerative diseases lead to enhanced or diminished endogenous NSC function.

Importantly, intrinsic and extrinsic changes associated with aging do not occur in isolation, rather they interact synergistically. For example, changes in diet are reflected in fluctuations of physiologic factors in the CSF, such as IGF-1 and insulin, which in turn contributes to intrinsic epigenetic regulation of NSCs (Mihaylova et al., 2014; Mir et al., 2017). Further, cell-intrinsic aging, which is reflected in senescent NSCs, alters the extrinsic microenvironment *via* the activity of the SASP.

SASP then leads to an amplification of local cellular dysfunction as SASP factors spread to and impair the normal functioning of local cells which in turn leads to further cell-intrinsic changes and beginning the cycle anew (Coppé et al., 2010).

Therefore, NSC aging cannot be attributed to a single factor, rather it involves a complex interplay of both intrinsic, including DNA damage, and extrinsic factors, such as secretion of inflammatory factors, which all contribute to dysfunction alterations in the stem cell niche.

## Modeling Brain Aging *in vivo*

Studying brain aging *in vivo* is a challenging endeavor given that the regulation of age is heterogenous amongst different tissues and species (Zahn et al., 2007), however, research has primarily focused on evolutionarily conserved mechanisms that control aging to better facilitate data extrapolation to humans. Numerous mouse models of aging have been established and extensively reviewed (Yeoman et al., 2012; Heng et al., 2017; Folgueras et al., 2018). Nevertheless, the field is still in its infancy, and major gaps in our knowledge exist regarding how the aging process impacts the central nervous system (CNS), specifically NSC function and maintenance. Here, we are going to briefly cover findings from mouse models of accelerated aging in which an NSC phenotype has been described.

Mouse models recapitulating aspects of genomic instability associated with aging have been established and extensively used to study this biological process in non-CNS tissue as reviewed elsewhere (Specks et al., 2015; Folgueras et al., 2018). Many mouse models of aging have been generated based on genetic correlates of progeroid human syndromes. Progeroid syndromes are a group of genetic disorders that make individuals biologically older than they are, typically due to a monogenic mutation in DNA repair mechanisms or lamin (Gordon et al., 2014). However, there are some mouse models based on progeroid syndromes that do not exhibit an NSC dysfunction, one example being a mouse model Hutchinson-Gilford progeria syndrome (HGPS). HGPS is caused due to a mutation in the lamin A gene, resulting in the build-up of the truncated form of lamin A, called progerin. Despite lamin A being ubiquitously expressed by various cell types, induced mutation of *LMNA* in the brain of mice did not affect behavior or neurogenesis assays of hippocampal cells, suggesting a protective mechanism (Baek et al., 2015). However, there are other relevant mouse models of aging that possess an NSC phenotype which will be further discussed.

### Benzimidazole-Related 1 (BubR1) Mutants

BubR1 is a spindle assembly checkpoint kinase, encoded by *Bub1b*, that is critical for the accurate separation of duplicated chromosomes during cell division and maintaining genomic stability (Wassmann and Benezra, 2001; Cleveland et al., 2003). Reduced BubR1 expression induces aneuploidy, which affects genomic stability (Baker et al., 2004). Further, deficiencies in DNA repair pathways cause progeria syndromes in humans which display CNS pathologies (Gordon et al., 2014). Natural aging significantly reduces BubR1 expression within the DG of the hippocampus in an age-dependent manner, with the largest reductions observed at 24 months of age. Interestingly, it only takes 2 months for BubR1 insufficient mice (*BubR1*^H/H^) to reach a similar expression level to that of wild-type mice at 24 months of age. NSC proliferation and maturation were significantly reduced in *BubR1*^H/H^ mice impairing hippocampal neurogenesis despite having no impact on neuronal survival (Figure 2; Yang et al., 2017). An independent study identified a similar impairment in NSC proliferation following lentiviral knockdown of *BubR1* in wild-type adult mice, confirming the strength of the *BubR1*^H/H^ mouse model in recapitulating aspects of NSC aging. Further, a follow-up study using the *BubR1*^H/H^ mice found that inhibition of the endogenous Wnt antagonist, secreted frizzled-related protein 3 (sFRP3), rescued NSC proliferation in the hippocampal DG in these mice (Cho et al., 2019).

These data highlight how mouse models of genomic stability can be employed to not only rapidly and faithfully recapitulate specific hallmarks of aging *in vivo* but allow for a mechanistic understanding of the aged NSC phenotype.

### Telomerase Mutants

Telomerase-deficient mice have served as a model system to study the adverse cellular and organismal consequences of wide-spread endogenous DNA damage signaling activation *in vivo*. In these mice, telomere loss and uncapping provoke progressive tissue atrophy, stem-cell depletion, and impaired tissue injury responses (Sahin and Depinho, 2010). Telomere attrition in NSCs is of particular interest as loss of proliferative potential and stem cell exhaustion with age is thought to be correlated with the age-related cognitive decline observed in mice and humans (Ferrón et al., 2009). Currently, there are two established telomere attrition mouse models with an associated NSC phenotype.

In one mouse model, mice carrying a homozygous germline deletion for the telomerase RNA component gene (*Terc*^−/−^) showed a complete loss of *Terc* expression and telomerase activity (Blasco et al., 1997). Inducing telomere shortening in these mice requires successive generations (>3 generations) of mating to establish phenotypic changes (Blasco et al., 1997). Third generation *Terc109:563*^−/−^ mice show reduced neurogenesis in the SVZ of the DG and loss of neurons in the hippocampus and frontal cortex, suggestive of an impairment in the proliferation and maturation capacity of NSCs (Ferrón et al., 2004; Rolyan et al., 2011; Khan et al., 2015).

In the other mouse model, deficiency in telomerase reverse transcriptase (*Tert*^−/−^), a catalytic subunit of the telomerase complex, induces aging phenotypes similar to that observed in *Terc*^−/−^ mice, such as impaired spatial memory, dendritic development, and neurogenesis (Strong et al., 2011;Zhou et al., 2017).

Interestingly, in a tamoxifen-inducible *Tert*^−/−^ mouse model (TERT-ER) somatic telomerase reactivation reversed these observed deficits through the restoration of proliferating Sox2^+^ NSCs and new-born neurons, consistent with the integral role of SVZ NSCs in the generation and maintenance of mature neurons in adulthood (Jaskelioff et al., 2011).

Thus, these transgenic mice provide excellent models for the study of the deficits in proliferation observed in NSCs of aged animals.

### Mitochondrial DNA Mutants

It is well accepted that mitochondria play a central role in the maintenance of NSCs in development and throughout adulthood, with oxidative phosphorylation an essential regulator of NSC proliferation and differentiation (Cabello-Rivera et al., 2019; Khacho et al., 2019). Consequently, age-associated alterations in mitochondrial function are thought to have a profound effect on the ability of NSCs to self-renew (Khacho et al., 2019). Mutations in the mitochondrial genome increase with age (Vermulst et al., 2007) and are associated with an increase in the production of mitochondrial ROS, which are potent signaling molecules that can impair stem cell self-renewal capacity (Hämäläinen et al., 2015).

Mouse models of advanced aging accumulate mtDNA mutations (PolgA and Tfam), associated with reductions in quiescent NSCs and a loss of self-renewal in the adult SVZ (Ahlqvist et al., 2012; Beckervordersandforth et al., 2017b). Further, NSC-specific ablation of nicotinamide phosphoribosyltransferase (Nampt), a rate-limiting enzyme that salvages the essential metabolic cofactor nicotinamide adenine dinucleotide (NAD^+^) and the main source of NSC NAD^+^ required for cell-cycle progression, reduced the NSC pool and their proliferation in adult mice (Stein and Imai, 2014). Importantly, these phenotypes can be rescued *via* supplementation of N-acetyl-L-cysteine and nicotinamide mononucleotide in the PolgA and Nampt mouse models, respectively (Ahlqvist et al., 2012; Stein and Imai, 2014).

Therefore, mouse models of accelerated aging as a result of mitochondrial dysfunction can serve not only a basic research role but also in the development of therapies aimed at reversing these aging phenotypes.

### SAMP8 Mice

A non-transgenic mouse model of accelerated aging exists. SAMP8 mice are a specific strain of inbred mice that exhibit a senescence-accelerated phenotype, which could be used to study how intrinsic homeostatic mechanisms of NSCs change with age. This short-lived strain progressively develops learning and memory deficits, along with a multisystemic aging phenotype (Takeda, 2009). The mice develop increased oxidative stress and astrogliosis in the brain, along with an accelerated loss of the NSC pool associated with a rise in astrocyte differentiation (Figure 2; Soriano-Cantón et al., 2015).

Consideration must be taken when interpreting results from these mice as extensive astro- and microgliosis is observed in parallel with the deficits in NSCs (Soriano-Cantón et al., 2015). No current studies have investigated the potential impact activated astrocytes and microglia in the (accelerated) aging hippocampus have on resident NSCs. Therefore, these mouse models provide a valuable tool to not only study cell-specific effects of aging but also in untangling the cell-cell interactions of NSCs and resident (or peripheral) cells in the context of aging.

## Modeling Brain Aging in a Dish with Stem Cell Technologies

To model human brain diseases in a dish, many laboratories rely on induced pluripotent stem cells (iPSCs) rather than somatic aged brain cells, which are rare and difficult to obtain. Additionally, human post-mortem samples reflect cell states at the end stage of the disease, hindering the study of disease pathogenesis. To generate iPSCs, primary human cells, such as fibroblasts or blood cells, are reprogrammed to an embryonic-like state, similar to embryonic stem cells (ESCs), through the expression of Yamanaka transcription factors. iPSCs are then given cues to direct differentiation towards the mature cell of interest to model disease. Typically, iPSCs are used to model diseases with genetic origins, such as ALS (*SOD1* mutation), familial PD (mutations in *Parkin* and *PINK1*), and HD (*HTT* mutation), yet many sporadic forms of these and other diseases occur with increasing age, such as sporadic PD, ALS, and progressive MS (PMS; Niccoli and Partridge, 2012; Reeve et al., 2014). iPSCs generated with specific mutations and deletions reflective of the disease state are an invaluable tool for understanding how familial genetic alterations change cell phenotype. However, the consideration of aging signatures is rarely considered.

Mimicking age-related defects in cells has proven difficult as reprogramming acts as a *developmental reset*, removing some of the known aging markers that were evident in the somatic cell of origin (Studer et al., 2015). These markers include epigenetic patterns, DNA damage, mitochondrial dysfunction, telomere length, and senescence, which are diminished or absent either during iPSC induction and/or further differentiation into the cell of interest (López-Otín et al., 2013).

The following reprogramming into iPSCs, several age-associated markers (including genetic and proteomic signatures) were reset, and cells retained a youthful phenotype (Miller et al., 2013). Despite this evidence, it is still unclear to what extent markers of the cell of origin are retained as it has also been shown that reprogrammed iPSCs can exhibit differentiation biases when further differentiated into a cell type of interest. This is of particular importance not only for cells in disease models but identifying what constitutes an aging signature vs. a disease-related signature. For example, NSCs differentiated from iPSCs reprogrammed from primary progressive MS (PPMS) patient fibroblasts and primary blood mononuclear cells exhibit a senescence phenotype compared to age-matched healthy patients (Nicaise et al., 2019). PPMS NSCs secrete SASP factors *in vitro* and inhibit remyelination in an *in vivo* model of PPMS, which was attributed to a dysfunctional phenotype arising from intrinsic defects in NSC function (Nicaise et al., 2017). Whether the NSCs retained an epigenetic or genetic signature unique to PPMS disease during iPSC reprogramming is unknown (Nicaise et al., 2019). This may also indicate that labile disease-associated markers which are lost with reprogramming are not necessary to recapitulate salient features of the disease or aging in the reprogrammed cells, therein pointing to the unmodified processes as playing a more important role in the preservation of “age” or disease underlying the utility of these cellular models. In support of these findings, a higher frequency of age-related mtDNA mutations is identifiable in iPSCs reprogrammed from fibroblasts of aged individuals compared to reprogrammed fibroblasts from young individuals. This work demonstrates that cellular reprogramming does not completely remove epigenetic markers nor the propensity of aged cells to accumulate mutations (Kang et al., 2016). Nor does iPSC reprogramming abrogate the potential for the study of idiopathic forms of the disease, as iPSC-derived astrocytes from non-genetic forms of ALS recapitulate the disease-related phenotypes of genetically confirmed ALS iPSC-astrocytes (Haidet-Phillips et al., 2011). Overall, these studies raise further questions regarding the conservation of age-related changes under disease conditions or whether disease conditions exacerbate the expression of markers of cellular aging (Table 1).

**Table 1:** List of studies using human stem cells to study cellular brain aging and disease.

Cell type	Donor ages (years)	Disease	Phenotypes and markers	Reference
iPSCs	24–72	None	Increased frequency of mitochondrial DNA mutations with age.	Kang et al. (2016)
iPS-NSCs	45–66	PPMS	Displayed senescence markers, secreted pro-inflammatory factors inhibiting OPC differentiation.	Nicaise et al. (2019)
hES-NSCs treated with hydroxyurea	Embryonic	None	Exhibited cellular senescence, impaired mitochondrial metabolism.	Daniele et al. (2016)
iNSCs	30–60	None	Cells retained 5–30% of donor cells epigenetic aging markers.	Sheng et al. (2018)
iPS-neurons with progerin overexpression	11–96	PD and HGPS	Dendrite degeneration, loss of TH, increased DNA damage and mitochondrial ROS.	Miller et al. (2013)
iPS-neurons treated with telomerase inhibitor	N/A	PD	Increased DNA damage, reduction in dendrites, increased mitochondrial ROS.	Vera et al. (2016)
iNs	0–89	None	Transcriptomic aging, loss of nucleocytoplasmic organization.	Mertens et al. (2015)
iNs	6–71	HD	HD gene expression increased DNA damage and ROS production, protein aggregates.	Victor et al. (2018)
iNs	0–89	none	Mitochondrial dysfunction—impaired energy production, lower mitochondrial membrane potential.	Kim et al. (2018)
iNs	37–71	ALS	Soma shrinkage, hypoactivity.	Liu et al. (2016)

*Abbreviations: ALS, amyotrophic lateral sclerosis; HD, Huntington’s disease; hES, human embryonic; HGPS, Hutchinson-Gilford progeria syndrome; iNs, induced neurons; iNSCs, induced neural stem cells; iPS, induced pluripotent stem; NSC, neural stem cell; OPC, oligodendrocyte progenitor cell; PD, Parkinson’s disease; PPMS, primary progressive multiple sclerosis; ROS, reactive oxygen species; TH, tyrosine hydroxylase*.

There is no universally agreed upon and established metric for determining cellular age, however, DNA methylation patterns are observed to correlate with chronological age, termed “epigenetic age.” One model which uses DNA methylation patterns is named Horvath’s model and is based on 353 CpG methylation sites in cells and tissues that allow for the quantifiable measurement of cellular biological age as well as an estimation of age acceleration (i.e., contrasting DNA methylation age with chronological age; Horvath et al., 2012). The specific methylation sites correlate to 0 years of age in ESCs, are reflected in somatic cells as the age of the donor but become largely erased upon iPSC reprogramming (Horvath et al., 2012; Horvath, 2013; Sheng et al., 2018).

Instead, direct induction of somatic cells to induced NSCs (iNSCs), retains 5–30% of the original aged methylation pattern according to the Horvath model, making them an ideal candidate to study aging both in the context of health and disease (Sheng et al., 2018). Direct induction of cells bypasses the pluripotency step, wherein somatic cells are forced to express the Yamanaka factors transiently then cued to become NSCs using growth factors *in vitro* (Thier et al., 2012). Recent work profiled markers of aging in young and aged blood cells, as well as iPS-NSCs and iNSCs from the same patients, and somatic fetal NSCs. Blood cells from aged patients match the estimated age based on Horvath’s model and possess shortened telomeres and an aged transcriptome, which was absent in the iPS-NSCs. Rather, iNSCs retain specific methylation patterns observed in the blood cells and shortened telomere length, while iPS-NSCs closely match the somatic fetal-derived NSCs (Sheng et al., 2018; Table 1). Whether these DNA methylation patterns are reflective of aging within cells or causative transcriptomic changes inducing cellular dysfunction is unknown.

The forced expression of neurogenic transcription factors in fibroblasts results in the generation of directly induced neurons (iNs), which are further differentiated *in vitro* to obtain a more mature neuronal subtype (Vierbuchen et al., 2010).

Induced motor neurons (iMNs) from primary fibroblasts or iPS-NSCs retain many of the aging hallmarks of the old donors, including increased DNA damage foci (γH2AX), SA-β-galactosidase activity, and loss of heterochromatin and nuclear organization (Tang et al., 2017). Aging signatures in iMNs derived from iPS-NSCs were largely erased. Further work using both iPSC-derived neurons and iNs from aged patients identified that decreased Ran-binding protein 17 (RanBP17) in aged iNs is responsible for the age-dependent loss of nucleocytoplasmic organization, which is reset in iPSCs (Mertens et al., 2015). Also, mitochondrial dysfunction present in the somatic cell is exacerbated in aged iNs, supporting the theory of metabolic dysfunction driving brain aging (Hoffman et al., 2017; Kim et al., 2018; Table 1).

The culmination of this work highlights the power of direct induction in disease modeling to further our understanding of the processes underlying age-related deficits in cells (Liu et al., 2016; Nekrasov et al., 2016). Unfortunately, caveats remain with this technology, such as the glaring fact that neurons are post-mitotic, restricting their use in large-scale applications, such as drug screens, due to the lack of a proliferative intermediate cell. However, their value lies with the insight into understanding neuronal aging in the context of health and disease. In the future it will be important to explore how disease-associated and aging phenotypes can be retained in iNSCs, and if direct induction into other brain cell types, such as astrocytes and oligodendrocytes, will uncover unknown roles of these cells in aging and disease.

Despite iPSCs losing some markers of aging during the reprogramming process, they are a valuable tool that can generate, theoretically, endless amounts of specialized cells for researchers to investigate. Some genetic mutations have been found to accelerate aging, including point mutations within the lamin A (*LMNA*) gene, which was identified in HGPS. Modification of the *LMNA* transcript results in a truncated form of lamin A called progerin. Progerin acts to shorten telomeres, inflict DNA damage, and encourage cellular senescence. Interestingly, iPSCs from patients with HGPS do not show an accumulation of progerin and lack the cellular alterations associated with accelerated aging, however, this phenotype is reinstated following iPSC differentiation into smooth muscle cells (Liu et al., 2011). This system was applied to iPSC-derived neurons from PD patients to determine the effect of aging on this disease. The researchers transfected progerin into iPSC-derived dopaminergic (DA) neurons from both healthy and genetic PD subjects and discovered disease-specific phenotypes. Progerin transfected PD cells developed an aging phenotype displayed by the progressive loss of tyrosine hydroxylase (TH) expression, enlarged mitochondria, and Lewy-body-precursor inclusions compared to healthy cells transfected with progerin, despite these cells still displaying accelerated aging signatures such as DNA damage foci (Miller et al., 2013). This work indicates that forced progerin expression can induce age-associated marker expression, but it is unknown if this method faithfully recapitulates cellular phenotypes observed in advancing age and disease.

Hydroxyurea (HU), used for the treatment of leukemia, disrupts cellular proliferation, and has been found to induce senescence in human cells (Park et al., 2000). Treatment of rodent NSCs *in vitro* with (HU) robustly induces cellular senescence identified by increased p53 and p16^Ink4a^ expression, SA-β-galactosidase staining, and downregulation of DNA repair proteins, which has also been replicated in human NSCs *in vitro* (Daniele et al., 2016). This model led to the formation of aging hallmarks through DNA damage, similar to ROS-induced damage in the brain, yet it is unknown if it faithfully recapitulates every aspect of aging, including telomere shortening and Horvath DNA methylation patterns (Dong et al., 2014; Table 1). HU treatment of NSCs derived from disease models, either directly induced or from iPSCs, has yet to be investigated but may yield interesting results.

Ionizing irradiation, which causes severe DNA damage and inhibits the proliferation of NSCs, is used to study senescence *in vitro*, however, it is unknown how well this faithfully recapitulates cellular hallmarks of aging (Schneider, 2014).

The aforementioned studies, which are summarized in Table 1, describe models that recapitulate some, but not all, of the key characteristics of cellular aging. However, each model comes with its own set of limitations. Thus far, iNs retain the largest number of aging markers identified within the parent cell, including maintenance of DNA methylation age, transcription of aging-associated factors, and mitochondrial dysfunction. Unfortunately, iNs do not capture the entire gestalt of the aging brain, nor do they have a proliferative intermediate to derive other cell types, but they are ideal for studying neurons in the context of diseases of aging. Their use in modeling aging and age-related disease is reviewed in depth elsewhere (Mertens et al., 2018). iNSCs may prove more useful for further interrogation of aging in disease as iNSCs are known to retain 5–30% of age-related DNA methylation markers from the cell of origin and have the capability to differentiate into astrocytes, oligodendrocytes, and microglia when prompted. iPS-NSCs from patients with PPMS have shown a senescent phenotype, however, iNSCs have yet to be used to model any age-associated disease. Forced expression of verified aging-associated proteins and treatment with inducers of senescence are viable alternatives in creating cellular aging hallmarks. Therefore, induced cellular aging may serve as a method to study how certain proteins or extrinsic factors affect pathways related to cellular aging in specific cell types, but their faithfulness in capturing the natural aging and disease process is debated. Since aging is a multifactorial process, applying a multipronged approach (i.e., disease based iNSCs or iPS-NSCs treated with cellular stressors) will help further elucidate new pathways and enhance our understanding of aging and disease. As such, these models may be especially useful in the sporadic forms of diseases, such as MS, PD, AD, and ALS, where no obvious genetic causes exist, or affect more than one cell type in the brain and involve aging.

## Novel Therapeutics for Brain Aging

With the emerging picture of experimental and pathological data pointing to disease-associated aging among progenitor cell populations, how might this be targeted for therapeutic benefit? Certainly, approaches such as caloric restriction and lifestyle can influence the risk of disease, but for some conditions, a patient’s ability or compliance for these approaches would also represent a challenge. Hence, pharmacological approaches to affect the aged cells within tissues are being actively explored as viable strategies to limit disease progression and/or stimulate endogenous regeneration and recovery (Table 2).

**Table 2:** List of novel therapeutics for brain aging.

Class	Definition	Drugs/Agents available	Mechanism of action	Citations using therapeutics on CNS cells
Senomorphics	Compounds that actively alter the physiology of senescent cells.	Metformin, rapamycin	Metformin—acts on Akt to inhibit mTOR Rapamycin—inhibits mTOR	Neumann et al. (2019) and Nicaise et al. (2019)
Senolytics	Compounds that actively kill senescent cells.	A combination of dasatinib (D) and quercetin (Q), fisetin	D—an inhibitor of tyrosine kinases. Q—inhibits PI3K Fisetin—inhibits mTOR	Currais et al. (2018) and Zhang et al. (2019)
NSC Transplantation	NSCs grafted into CNS.	Allogenic mouse NSCs, NSCs with neurotrophic supplementation.	Cell replacement strategy, secrete beneficial factors.	Park et al. (2013) and Zhang et al. (2017)

*Abbreviations: CNS, central nervous system; mTOR, mammalian target of rapamycin*.

Drug screens using NSCs are not as common as disease-specific cell types such as oligodendrocytes for MS or neurons in diseases such as AD and PD. However, when considering the potentially influential role of senescent NSCs in the context of impaired regeneration or functionality, the paucity of high throughput or high content screens using rodent NSCs, or disease-specific NSCs, represents a potential opportunity for therapeutic interventions for a range of neurological conditions. Conceptually, the effect of NSCs as a source of disease-related impairment would mean that drug screens using NSCs would not only impart effects for neurogenesis but potentially also a means to address microgliosis, astrogliosis, and promote myelin regeneration (Kim and Jin, 2012). In terms of the latter, targeting cellular senescence in NSCs could represent a means by which to affect cognitive decline which is linked to reduced myelination with advancing age (Wang F. et al., 2020). Similarly, the diminished neuroendocrine function of the hypothalamus is associated with the cognitive decline with advancing age and a consequence of loss of hypothalamic NSC functions (Zhang et al., 2013). This loss of function is a result of the NSC senescence of hypothalamic NSCs (Xiao et al., 2020).

Using primary neurospheres of murine hypothalamic NSCs, the long non-coding RNA Gm31629 was found to be reduced with aging and function as a biomarker of cellular senescence through a stabilized interaction with Y-box1 (YB-1) protein (Xiao et al., 2020). Based on this, nuclear magnetic resonance (NMR) was applied to understand how Gm31629 conferred stability to YB-1 to then perform a molecular docking and virtual screen of a library of natural small molecule compounds that identified theaflavin 3-gallate (TF2A; Xiao et al., 2020). This compound, a theaflavin found in black tea, was found to reverse senescence-like changes in senescent cells, and administration to 12-month-old mice reduced the rate of age-related decline (Xiao et al., 2020). These findings prompt several conclusions: (i) NSCs are an important site of cellular senescence; (ii) NSCs can exert widespread functions in aged animals throughout the body; and (iii) NSCs can be effectively used to screen and identify anti-senescence agents. From these data, future screens employing human disease-specific NSCs would be warranted to address the role of cellular senescence in this population plays in contributing to neuropathology and disease progression.

Further screens of existing chemical libraries have yielded nomenclature to describe two categories of senotherapeutic agents, termed “senomorphics” and “senolytics” (Table 2).

### Senomorphics

Senomorphic agents are compounds that actively alter the physiology of aged cells, thereby mitigating their influence on the cellular environments in which they reside (Table 2; Tchkonia et al., 2013). Specifically, these compounds disrupt or block the production and release of pro-inflammatory factors that comprise the SASP (Farr and Khosla, 2019). Senomorphics thereby block the paracrine effects of senescent cells on other cells, limiting the impact of the SASP on impaired tissue function and/or regenerative potential. Examples of senomorphic agents which have been explored in the context of aging in demyelinating disease include metformin and rapamycin. Of particular relevance to our discussion on NSCs is the recent report demonstrating the utility of metformin to enhance the myelinating capacity of OPCs in the aged CNS (Neumann et al., 2019). Rapamycin is a naturally occurring macrolide product first isolated in 1931 from a bacterium found in the soil on Easter Island (Rapa Nui) in the south Pacific Ocean (Vézina et al., 1975). The anti-inflammatory actions of rapamycin are mediated by the kinase mTORC1 (Brown et al., 1994). Similarly, metformin acts *via* Akt (PKB) to inhibit mTOR signaling (Dowling et al., 2007). Interestingly, a CRISPR-Cas9 knockout gene screen of senescent cells identified *MTOR* as a critical checkpoint required for the SASP (Liu et al., 2019), in part, *via* attenuated nuclear factor kappa-light-chain-enhancer of activated B cells (NFkB) signaling and suppressed IL-1RA levels (Laberge et al., 2015; Liu et al., 2019). Consistent with these findings, we had recently determined that NSCs derived from MS patient iPSC lines exhibit a cellular senescence phenotype and upregulated mTOR expression. Treatment of these patient NSCs with rapamycin was similarly found to both reduce mTOR expression and the senescent phenotype in this cellular model (Nicaise et al., 2019).

### Senolytics

Senolytic agents represent the second class of agents to therapeutically address the impact of cellular aging in intractable or incurable diseases. The compound term senolytic refers to the mechanism of action of this class which is to selectively kill cells that are senescent to reduce the impact of these cells on tissues (Table 2). This novel class of agents was named by Zhu et al. (2015). Using irradiated fat cells, they validated six anti-apoptotic targets by siRNA, which included B-cell lymphoma-extra large (BCL-XL) and ephrin type-B receptor 1 (EPHB1), that were found to promote senescent cell death but did not affect the viability or proliferation of non-senescent cells. From this, a limited biased screen of select compounds identified the combination of dasatinib (D), an SRC-family protein-tyrosine kinase inhibitor approved for the treatment of leukemia, and quercetin (Q), a dietary flavonoid with estrogen mimetic activity and pleotropic kinase inhibitor (Russo et al., 2014), were found to be most effective in clearing/killing senescent cells (Zhu et al., 2015). Other agents in the senolytic class, such as fisetin (Zhu et al., 2017), which mimics the effects of dietary restriction, are also in development for their potential to target senescent cells in disease models including CNS conditions (Currais et al., 2014, 2018). Characterization of the impact of these agents on the CNS requires more extensive study, as it is unknown to what extent killing senescent cells would alter the brain microenvironment. A potentially important caveat to the application of these agents to CNS disease is that in many instances the molecular basis for understanding cellular senescence is from irradiated (10 Gy) non-neural primary cells such as fibroblasts or fat cells. Although these studies and screens provide an important molecular template for identifying cellular senescence in other tissues, including CNS, the precise translation of changes evoked by irradiation to disease conditions within the CNS may be incomplete and likely there exist important differences requiring further characterization and study.

Experimental evidence from transgenic animal models has shown the potential utility of senolysis as an effective strategy to target senescent cells in the CNS *in vivo*. Currently, experimental models of AD are being tested as prototypic neurological diseases of aging (Mendelsohn and Larrick, 2018). For example, Baker et al. (2011) developed a transgenic mouse in which senescent p16^Ink4a+^ cells are killed by the initiation of apoptosis through chemically included expression of ATTAC, a caspase inducer sequence. They recently reported that astrocytes and microglia develop an aged, p16^Ink4a+^ (senescent) phenotype in a presenilin mouse model of AD-related tauopathy (MAPT^P301S^PS19; Yoshiyama et al., 2007). Then, using the INK-ATTAC transgenic mice, they demonstrated that selective elimination of these p16^Ink4a+^ cells improves the phenotype and amyloid load in a MAPT^P301S^PS19 mouse model of tau-dependent AD-like disease (Bussian et al., 2018). Importantly, this study used electron microscopy to show that neurons do not develop a senescent-like phenotype. This was indicative that cells with proliferative potential may be key players in neurological illnesses associated with aging (Bussian et al., 2018), whereby microglia play an important role in shaping neuropathology. Consistent with this transgenic study, the identification of senescent-like changes in p21+ and p16^Ink4a+^ OPCs is associated with amyloid plaques in human AD autopsy neurospecimens and in an APP/PS1 mouse model of AD (Zhang et al., 2019). They also demonstrated that the use of the senolytic combination D + Q effectively eliminates the abundance of senescent cells, which was coincident with a dramatic improvement of cognitive performance and lowered amyloid load in treated APP/PS1 mice (Zhang et al., 2019). Similarly, the senolytic fisetin has been reported to ameliorate cognitive deficits in APPswe/PS1dE9 AD mice (Currais et al., 2014). Hence, accumulating evidence supports a role for cellular senescence associated with cognition and neuroinflammation in AD. The impact of these agents on cognitive decline in aged individuals without associated neurological diagnosis or other neurodegenerative diseases will require further study.

### Cell Transplantation

NSCs have shown promising effects as exogenous stem cell therapeutics in the treatment of many mouse models of neurodegenerative diseases, such as PD, AD, MS, and ALS (Martino and Pluchino, 2006; Pluchino et al., 2009; Willis et al., 2020). Major strides have been made in harnessing their restorative, regenerative, and protective potential in the hopes of treating these devasting diseases. NSC transplants not only act as a cell replacement strategy but also secrete factors that can promote endogenous regeneration (Table 2). However, if their usefulness becomes diminished in an aged milieu is unknown.

An important caveat in cell transplantation is the idea of host-to-graft transfer of disease, which has been observed in PD patients. Foetal-derived NSCs transplanted into patients were found to last up to 16 years but eventually developed alpha-synuclein-positive Lewy bodies (Li et al., 2008). This work stresses the importance of the extracellular milieu and how it can affect even NSCs of fetal origin. As described in the above section, there is a myriad of extrinsic aging factors that would affect the transplanted graft. For example, the increase in pro-inflammatory factors, as well as the build-up of dysregulated proteins may outweigh the benefit of the transplanted cells and affect the overall success, possibly even turning transplanted cells senescent. Early work points to the success of fetal NSC transplants into aged rats, resulting in cognitive improvements. However, cells transplanted into aged and damaged environments demonstrate lower graft survival (Winkler, 2001; Zaman and Shetty, 2002). Interestingly, combining neurotrophic supplementation of brain-derived neurotrophic factor (BDNF) and neurotrophin-3 (NT-3), along with a caspase inhibitor, allows the graft to survive. Genetically engineered NSCs, which are resilient to inflammation (expressing dominant-negative nuclear factor of kappa light polypeptide gene enhancer in B-cells inhibitor, alpha [IκBα]), display an improvement in survival after transplantation into aged mice (Zhang et al., 2017). Not only can cells be engineered to be more resistant to hostile conditions, but they can also be genetically altered to secrete increased growth factors and even enzymes, such as choline acetyltransferase (ChAT), which is found to improve cognitive function in aging animals (Park et al., 2013). These examples suggest the idea of a multifaceted approach where young NSCs can be harnessed to secrete pro-rejuvenating and neurotrophic factors, such as TIMP-2, to help promote endogenous niche activation and eventually brain regeneration in aging and disease (Castellano et al., 2017). This method could potentially be used in conjunction with a senomorphic approach to have the most effect. However, as noted above, to what extent young NSCs or genetically modified NSCs remain healthy and unperturbed by the aged environment is unknown. Over time the chronically inflamed brain may confer age-related changes onto the transplanted cells rendering them as dysfunctional as the endogenous niche. Stem cell therapy for neurodegenerative disease and aging may hold the promise of regeneration, nevertheless, many hurdles remain.

## Conclusion

Research on brain stem cell aging within the past decade has surged and we have made significant advances in understanding what happens to this cell population in aging and disease using a variety of *in vitro* and *in vivo* model systems. Intrinsic and extrinsic factors play a large role in the decline of the NSC niche over time, which can be exacerbated by the disease. These include DNA damage, changes in metabolism and nutrient sensing, as well as the chronic pro-inflammatory environment both within the niche and in systemic fluids, which can all act to maintain cells in either a quiescent or senescent state, inhibiting their proper function when needed. These putative markers of NSC aging may also lead to the identification of biomarkers predictive for the onset and/or progression of cognitive defects associated with advancing age and neurodegenerative diseases. For example, the blood-borne SASP proteins IL-15 and chemokine (C-C motif) ligand 3 (CCL3) are correlated with chronological age and cognitive decline (Lehallier et al., 2019; Schafer et al., 2020). However, the cellular sources of these SASP factors are currently unknown.

Therefore, using patient-derived NSCs generated from fibroblasts and profiled for senescence markers may identify the proportion of circulating proteins contributed by NSCs. Further study is needed to determine if the NSC senescence burden *in vitro* correlates with the development and progression of cognitive defects *in* *vivo*.

There is no single mechanism to aging, and multiple processes likely contribute to declining. Therefore, using models in which we can parse these details is important to understanding the fundamentals of this process. Interrogating how aging may differ from diseases associated with aging, both transcriptionally and proteomically, would allow us to better understand the discrepancy between those who do and do not develop the disease, but also identify what facets of aging portend disease. This includes *in vivo* models of aging using genetically altered mice in which certain genes are mutated affecting various age-associated pathways (i.e., mitochondrial dynamics), allowing researchers to study in detail how changes alter cellular and organismal aging. *In vitro* models using novel stem cell technologies also hold potential for studying NSC aging in humans as they retain aging and disease markers. Defining the mechanisms that trigger modifications in this cell population with age and in disease will allow for a more directed approach to developing preventative or curative treatments.

## Author Contributions

AN designed review outline, wrote manuscript, and designed figures. CW and SC contributed sections of manuscript. CW, SC, and SP critically reviewed manuscript.

## Conflict of Interest

SP is co-founder and CSO at CITC Limited and iSTEM Therapeutics, and co-founder and Non-executive Director at Asitia Therapeutics. AN is an advisor for iSTEM Therapeutics. The remaining authors declare that the research was conducted in the absence of any commercial or financial relationships that could be construed as a potential conflict of interest.
